# Workplace mistreatment of Swedish health care professionals: prevalence and perpetrators across profession, sex, and birth country

**DOI:** 10.1186/s12913-025-12620-0

**Published:** 2025-03-29

**Authors:** Britta E. Gynning, Elinor Forsheden Sidoli, Katrina J. Blindow, Emma Cedstrand, Erika L. Sabbath, Emma Brulin

**Affiliations:** 1 The Department of Occupational Medicine, Institute of Environmental Medicine, Karolinska Institutet, Nobels väg 13, Solna, 171 65 Sweden; 2 Center for Occupational and Environmental Medicine, Region Stockholm, Solnavägen 4, Stockholm, 11365 Sweden; 3 Harvard T.H. Chan School of Public Health, Harvard University, 677 Huntington Ave, Boston, MA 02115 USA

**Keywords:** Mistreatment, Workplace incivility, Sexual harassment, Gender-based harassment, Ethnicity-based harassment, Nurses, Physicians

## Abstract

**Background:**

This study explores the prevalence and co-occurrence of workplace mistreatment of nurses and physicians and their perpetrators. We explore the distribution of incivility and identity-based harassment (ethnicity-, gender-based, and/or sexual harassment), combined with sex and birth country in these two professional groups.

**Methods:**

This cross-sectional study draws on survey data, including an analytical sample of 141,237 Swedish nurses and physicians in 2022.

**Results:**

Incivility was the most prevalent mistreatment type, ranging from 28.2% among male physicians to 59.5% among nurses born outside Europe. Identity-based harassment ranged from 18.1% among male physicians to 57.8% among nurses born outside Europe. Incivility stemmed most commonly from internal perpetrators (co-workers), and harassment was more commonly experienced by patients or their relatives.

**Conclusions:**

The mistreatment of healthcare professionals was widespread. Different types of mistreatment often co-occur, but prevalence varies substantially. The protective effect of professional status was dependent on social structures.

**Supplementary Information:**

The online version contains supplementary material available at 10.1186/s12913-025-12620-0.

## Background

Interpersonal mistreatment is a highly prevalent work environment problem and is widely recognised for its detrimental consequences both for the affected individuals and for organisations [1, 2]. Victims of workplace mistreatment have higher rates of outcomes such as burnout, depression, psychotropic medication use, and suicide [3–8]. Interpersonal mistreatment comprises a variety of counter-normative behaviours or actions with more or less overt discriminating content and intent to harm the target [2]. Although different kinds of mistreatment are often experienced together [9, 10], prior research has predominantly investigated single forms of mistreatment in isolation (e.g., sexual harassment or bullying) [11, 12]. In this study, we analyse the joint exposure to workplace incivility and identity-based harassment, i.e., ethnicity-, gender-based, and sexual harassment, as well as how these different kinds of interpersonal mistreatment relate to each other.

The healthcare sector is characterised by its hierarchical and gendered professions, patient contact, and a high degree of responsibility [13, 14]. Previous studies on the healthcare sector show that social hierarchies based on sex, ethnicity, and profession influence people’s risk of experiencing mistreatment in the workplace [1, 15]. Also, there is a need to better understand how different types of mistreatment, i.e., incivility and identity-based harassment, overlap and relate to each other [4, 9, 10, 12].

The relationships and power dynamics among health care professionals are distinct from their external relationships, i.e. with patients and visitors. This may influence the nature of mistreatment that occurs and the characteristics that put healthcare professionals at risk for adverse experiences. However, few studies have examined how sex and ethnicity shape healthcare providers’ experiences of workplace mistreatment, particularly concerning different perpetrator groups. To better understand the mechanisms behind the occurrence of workplace mistreatment in the healthcare sector and target interventions accordingly, it is crucial to explore how different social positions, as defined by sex, birth country, and professional status, are related to these experiences. Therefore, we study the prevalence and social patterning of interpersonal mistreatment by co-workers and patients separately.

This study makes a timely contribution by the utilisation of data, analysing two different professions, physicians and nurses, their experiences of four different types of mistreatment and how these types of mistreatment co-occur along with differences on an individual level of sex and birth country.

### Concept and definition of interpersonal mistreatment

Several concepts and definitions are used when studying interpersonal mistreatment. In this study, we explore two types of mistreatment, workplace incivility and identity-based harassment, defined as follows:

- *Workplace incivility* (WI) is impolite behaviour with ambiguous intent to harm another individual [5]. Workplace incivility violates norms for mutual respect and, unlike sexual or identity-based harassment, has no explicit discriminating or sexual content (ibid.). Persistent incivility towards a specific person can constitute a case of bullying. However, established bullying definitions involve a range of direct verbal and physical attacks and spreading rumours, which workplace incivility does not [16]. More subtle and ambiguous behaviours characterise workplace incivility [2], for example, condescending behaviour or comments, being ignored or unprofessionally met [4].
- *Identity-based harassment* is here used as an umbrella term for behaviours and actions that target people based on characteristics of their social identity or their membership in a group and can be verbal and physical. It is a form of discrimination that occurs on a personal level and includes behaviours that hinder the inclusion of affected individuals based on their social identity [4, 10]. EU law protects against workplace sexual harassment (Directive 2002/73/EC) and harassment due to sex, ethnicity, religion, sexuality, disability, and age (Directive 2000/43/EC; Directive 2000/78/EC). This study focuses on harassment based on ethnicity, gender and sexual harassment.

Ethnicity-based harassment (EBH) encompasses negative comments about a person’s ethnic background and their exclusion from work-related or social interactions because of their ethnicity [17]. Studies about harassment of people based on their birth country or ethnicity also use the term race-based harassment or racism [18, 19]. However, racism is a more comprehensive term, as it can be defined as a whole social system where people are divided and ranked based on physical characteristics, culture, and historical domination [13, 19].

Gender-based harassment (GBH) refers to experiences of disparaging conduct not intended to elicit sexual cooperation; instead, these are crude, verbal, physical and symbolic behaviours that convey hostile and offensive attitudes about members of one sex [20].

Sexual harassment (SH) is unwelcome conduct of a sexual nature that offends someone’s dignity and makes them feel uncomfortable, humiliated, intimidated, threatened, or hurt in their integrity [21, 22]. Sexual harassment can take different unwelcome physical, visual, verbal, or non-verbal forms, such as unwelcome compliments and invitations, groping, indiscreet stares, and showing of pornographic pictures, as well as offensive, sexually explicit digital contacts (ibid.).

### Prevalence of mistreatment and perpetrators

Studies about workplace mistreatment differ greatly in their prevalence estimates [23]. To some extent, this is explained by the diversity in concepts and definitions of mistreatment. Another reason could be that various types of mistreatment tend to co-occur [9], which is rarely taken into consideration in previous research.

One study, including different work sectors in the USA, found women and people of colour to experience more workplace incivility than men and white people, respectively [4]. The authors argue that, although not openly discriminating, workplace incivility may be directed selectively at women and people of colour, particularly racialised women, and constitute a subtle form of discrimination.

Employees often experience sexual harassment together with other forms of mistreatment, e.g., incivility and ethnicity- and gender-based harassment [10, 12]. Previous studies found that gender-based harassment and sexual harassment are closely related since unwelcome sexual behaviours are found to co-occur with non-sexualizing expressions of sexism in organisations [3, 9, 20, 21].

An international review recently highlighted that ethnic minority healthcare professionals report high victimisation by bullying from co-workers [19]. Similarly, Swedish studies reveal higher bullying rates among foreign-born nurses compared to their Swedish counterparts [24], especially those born in countries culturally different from Sweden [16]. Previous research in a Swedish healthcare context also found that ethnic minority healthcare staff reported experiencing racism and discrimination, highlighting a lack of space for discussion and reporting [18] and that medical students born outside of Europe faced the most common discrimination based on ethnicity [15].

A recent meta-analysis found higher levels of all types of mistreatment among women compared to men [25]. Velin et al. [15] showed that both male and female medical students experience some type of discrimination due to sex, age, sexuality, religion, or ethnicity. However, women experience it more: 50% of female students and 30% of male students reported that they experienced some type of discrimination [15]. Regarding workplace sexual harassment, studies show that both men and women are victims, but women are more frequently sexually harassed – especially young women [25, 26]. Yet, being a man in a female-dominated workplace increases the risk of being sexually harassed [27]. Studies have identified high rates of sexual harassment among healthcare professionals compared to other occupations [21, 28]. Also, Choo et al. [29] point out sexual harassment as a substantial organisational problem for female physicians.

Several studies show that in addition to being exposed to interpersonal mistreatment from co-workers, nurses and physicians are also exposed to mistreatment from patients and patients’ relatives [1, 15, 30]. Working in close physical contact with unclear boundaries between what patients are “allowed” to do can affect organisations’ response to sexual harassment [30, 31]. Prioritisation of patient satisfaction has been identified as an important risk factor for exposure to sexual harassment among health professionals [30], as well as racial slurs and microaggressions [18, 32]. Some healthcare professionals also perceive that unwelcome sexual behaviours are considered part of the job, and complaints from healthcare professionals are therefore dismissed [26, 30, 31]. A particular challenge in the healthcare context can be handling intrusive or offensive behaviours from patients with cognitive disabilities or disorders [30, 31].

### Hierarchical structures in health care

Healthcare systems are organised in hierarchal structures based on education and level of training, accentuating existing power structures [13, 14]. Harassment is often more present in workplaces with elevated levels of inequality and discrimination, including compensation, opportunity, and advancement [33]. In organisations with hierarchical work environments, sexual harassment may even be deemed more acceptable by those higher in the hierarchy [34]. Previous studies also show that more work experience, often linked to a higher hierarchical status in the organisation, protects individuals from workplace incivility [1]. In addition, results from a study at an Academic Medical Center in the USA indicate that the health impact of experiencing sexual and gender-based harassment may be buffered by the seniority of the victim [35].

## Aim and research questions

This study explores the prevalence and co-occurrence of different types of interpersonal mistreatment, i.e., workplace incivility and identity-based harassment (based on ethnicity or gender and sexual harassment) among nurses and physicians in Sweden. Furthermore, we aim to explore whether there are similarities and differences in perpetrator characteristics between these professional groups. The following research questions will be explored:

I. What is the prevalence, and who is the perpetrator of different types of mistreatment across professions, sex, and birth country?
II. To what degree do different types of mistreatment co-occur?

## Method

This study draws on data from the Longitudinal Occupational Health Survey for Health Care in Sweden (LOHHCS). LOHHCS includes a representative sample of practising nurses and physicians in Sweden in 2022. From the Swedish Occupational Register, a total of 7 790 nurses and 7 908 physicians were drawn using stratified random sampling. All sampled individuals received postal invitations from Statistics Sweden to participate in the study, including log-in information for a web-based survey. The response rate was 37.3% for nurses and 34.3% for physicians. Statistics Sweden also calculated calibrating weights to adjust for missing data, sampling errors, and stratification. The weights are applied in this study, resulting in an analytical sample of 141 237 individuals, of whom 102 685 nurses and 38 552 physicians.

This study was approved by the Swedish Ethical Review Authority (Ethical Review number 2021-05574-02 & 2022-00310-02).

### Measurements

#### Measurement of mistreatment

*Workplace incivility* was measured through the workplace incivility scale [36] including seven items measured using a 5-point Likert scale ranging from “Experiences every day” to “Never”. The scale included questions regarding experiences of humiliating or condescending remarks, insulting comments, or feeling ignored by a colleague. The seven items were compiled using a grand mean score ranging from 1 to 5 (Cronbach’s Alpha: 0.878).

*Ethnicity-based harassment*, *gender-based harassment*, and *sexual harassment* were measured through single items, and each rated on a 4-point Likert scale ranging from “Not exposed last 12 months” to “Exposed several times/weeks”. The questions were formulated: In the last 12 months, how often have you …*been harassed or discriminated against because of your ethnicity or skin colour?* (ethnicity-based harassment); …*been subjected to things that violated your privacy or were perceived as degrading because of your gender?* (gender-based harassment); …*been subjected to unwelcome advances or offensive remarks of a sexual nature (unwelcome advances or offensive innuendos about things generally associated with sex or your body)?* (sexual harassment). We combined ethnicity-based harassment, gender-based harassment and sexual harassment into one single variable indicating the experience of *identity-based harassment*.

Each measurement of mistreatment, workplace incivility, and identity-based harassment was thereafter dichotomised into no exposure versus any frequency of exposure. We created a composite variable to indicate experiences of mistreatment co-occurrence, including categories of experiencing no types of mistreatment, one type of mistreatment, or two or more types of mistreatment.

For every measurement of mistreatment, respondents could indicate the perpetrator’s professional role. The options included physician, manager, nurse, other co-workers, and patient/patient family, plus assistant nurses for nurses and consultants for physicians. Respondents could choose multiple perpetrators. Perpetrator identity was dichotomised into (i) internal perpetrator (co-workers within the health care sector, e.g., physicians, nurses, consultants, other co-workers) and (ii) external perpetrator (patients or their relatives).

#### Measurement of social structures in health care professionals

To compare between and within professions, we created two new variables stratifying professions by (1) sex and (2) birth country.

### Statistical analysis

The study first used descriptive statistics to characterise the sample. Frequencies were computed to address the first research questions, i.e., to explore the prevalence and sources of various mistreatments within different groups. We also used frequencies presenting the prevalence of each of the three identity-based harassments presented in the Supplemental files.

The second research question, which explored the co-occurrence of mistreatment types, was also addressed by tabulating frequencies.

Sensitivity analyses tested the combined effect of social structures (i.e., profession, sex, and birth country) on variations in the experience of different types of mistreatment using multilevel models. These models considered the influence of social structure groups (Model 1) and, additionally, of age groupings (Model 2) on the experience of mistreatment. The interclass correlation (ICC) was used to assess the impact of social structure groups along with age.

All analyses were conducted using SPSS version 28.0, and statistical weights were applied throughout.

## Results

### Study population

The study population (with weights applied) consisted of 144 237 individuals, 73% nurses and 27% physicians (Table 1). There were proportionally more men among the physicians (48.3%) than the nurses (11.3%). Regarding birth country, being born in Sweden was the most common, both among nurses (91.3%) and physicians (70.9%).

**Table 1:** Analytical sample of demographics by profession

	Total	Nurses	Physicians
	Count(%)	Count(%)	Count(%)
**Number of**	141,237(100)	102,685(72.7)	38,552(27.3)
**Sex**			
Women	109,697(78.0)	91,087(88.7)	18,610(51.7)
Men	31,540(22.0)	11,598(11.3)	19,942(48.3)
**Country of birth**			
Sweden	119,907(85.8)	92,983(91.3)	26,924(70.9)
Within Europe	13,026(9.3)	5305(5.2)	7721(20.3)
Outside Europe	6897(4.9)	3566(3.5)	3331(8.8)
**Sex by birth country**			
Sweden women	96,229(68.8)	82,601(81.1)	13,628(35.9)
Sweden men	23,678(16.9)	10,383(10.2)	13,295(35.0)
Europe women	9290(6.6)	4834(4.7)	4456(11.7)
Europe men	3736(2.7)	471(0.5)	3265(8.6)
Non-Europe women	4479(3.2)	3030(3.0)	1449(3.8)
Non-Europe men	2418(1.7)	536(0.5)	1882(5.0)
**Seniority**			
> 15y	66,900(47.5)	50,404(49.2)	16,496(42.9)
11-15y	23,507(16.7)	16,617(16.2)	6890(17.9)
5-10y	24,132(17.1)	16,474(16.1)	7658(19.9)
< 5y	26,303(18.7)	18,896(18.5)	7407(19.3)

Nurses’ ages ranged from 23 to 69, with a mean of 45.6 (Sd. 12.4) and a mode of 37. Physicians’ ages ranged from 25 to 76, with a mean age of 45.1 (Sd. 12.2.) and a mode of 37. Nearly half (47.5%) of all individuals in the study had over 15 years of experience as a nurse or a physician, with nurses having slightly less experience.

### What is the prevalence, and who is the perpetrator of different types of mistreatment across professions, sex, and birth country?

Table 2 presents data on the prevalence of mistreatment and the perpetrator, categorised by profession and stratified by sex, and birth country. Workplace incivility was the most prevalent form of mistreatment within the sample. Prevalence rates ranged from 28.2% among male physicians to 59.5% among nurses born outside Europe. Nurses, in general, reported a higher workplace incivility prevalence compared to physicians. Moreover, co-workers were more commonly identified as perpetrators of workplace incivility across all groups compared to patients or their relatives.

**Table 2:** Prevalence and perpetrator of workplace incivility and identity-based harassment across profession, sex and ethnicity (%)

			Nurses						Physicians					
				Sex		Birth Country				Sex		Birth Country		
		**Total Sample (%)**	**Total nurses (%)**	Women (%)	Men (%)	Sweden (%)	Within Europe (%)	Outside Europe (%)	**Total physicians (%)**	Women (%)	Men (%)	Sweden (%)	Within Europe (%)	Outside Europe (%)
**Workplace Incivility**	Incivility last 12 months	29.5	34.4	35.6	33.1	32.9	50.0	53.3	25.0	26.5	23.1	23.6	28.7	37.6
	Perpetrator^*^													
	Not stated	5.0	4.5	4.5	4.1	4.1	4.5	10.8	7.0	6.2	7.9	7.4	2.0	13.9
	Solely internal exposure	32.1	30.9	32.6	17.5	29.8	40.2	31.8	36.5	40.7	31.1	32.6	44.5	42.1
	Solely external exposure	8.1	8.1	7.7	11.2	7.8	8.7	13.5	8.3	5.4	12.2	8.9	8.1	5.1
	Internal and external exposure	54.8	56.6	55.3	67.1	58.3	46.5	43.9	48.2	47.7	48.8	51.1	45.4	38.8
**Identity-based harassment**	Identity-Based Harassment last 12 months	28.1	28.4	28.8	25.7	26.8	35.8	57.8	27.3	35.9	18.1	25.7	27.3	38.8
	Perpetrator^*^													
	Not stated	36.1	33.8	33.1	40.1	36.9	19.9	8.4	42.5	52.1	21.9	50.4	31.7	12.4
	Solely internal exposure	10.5	8.8	8.2	14.4	8.0	16.1	13.5	15.2	11.3	23.5	13.2	18.0	23.9
	Solely external exposure	43.5	48.3	49.3	40.1	48.1	53.2	48.9	29.9	27.0	36.1	29.7	26.3	37.8
	Internal and external exposure	9.9	9.0	9.4	5.4	7.0	10.8	29.2	12.3	9.5	18.5	6.7	24.0	26.0

^*^Of those with > = 1 experience in the past 12 months

For identity-based harassment, the prevalence ranged from 18.1% among male physicians to 57.8% among nurses born outside Europe. Female healthcare professionals generally experienced a higher prevalence than their male counterparts, with female physicians having the highest prevalence. Identity-based harassment from patients or relatives was more prevalent than harassment from co-workers, particularly among nurses.

Examining the prevalence of specific harassment types (see Supplemental table A2), gender-based harassment was prevalent across various stratified categories, with the highest prevalence (30.1%) observed among female physicians and the lowest among male physicians (8.3%). Ethnicity-based harassment, on the other hand, exhibited a clear incline based on birth country, most notably among individuals born outside of Europe, 50% of all nurses and 30% of all physicians born outside of Europe reported ethnicity-based harassment. Regarding perpetrators, notably, half of those having experienced ethnicity-based harassment were exposed by an external perpetrator (Supplemental Table A2); 20%, on the other hand, reported having experienced ethnicity-based harassment from another co-worker (internal perpetrator).

Sensitivity analysis (Supplemental Table A4) showed that social structures based on profession, sex, and birth country explained about 6% of the individual variation in the experience of workplace incivility and about 12% of identity-based harassment.

### To what degree do different types of mistreatment co-occur?

When examining the co-occurrence of mistreatment (Table 3), about half of the study participants experienced some form of mistreatment in the past year, with physicians experiencing it less frequently (39.9%) compared to nurses (47.4%). Table 3 shows the share of those who solely reported workplace incivility (16.8%), identity-based harassment (12.2%), or both (16.4%).

**Table 3:** Isolated mistreatment experience by profession and sex/birth group^*^

		Nurses						Physicians					
			Sex		Birth Country				Sex		Birth Country		
Mistreatment form	**Total experience (%)**	**Total nurses (%)**	Women (%)	Men (%)	Sweden (%)	Within Europe (%)	Outside Europe (%)	**Total physicians (%)**	Women (%)	Men (%)	Sweden (%)	Within Europe (%)	Outside Europe (%)
(1) Solely Incivility	16.8	18.6	18.5	18.6	18.2	28.3	12.7	12.0	10.8	13.3	12.3	10.5	14.0
(2) Solely identity-based harassment	12.2	11.4	11.7	9.3	11.1	13.3	18.7	14.1	19.1	8.6	13.9	11.9	19.4
(3) Incivility and identity-based harassment	16.4	17.5	17.4	16.9	16.2	23.1	39.7	13.8	17.3	9.8	12.2	15.8	20.4
(4) No incivility and no identity-based harassment	54.7	52.6	52.2	55.4	54.5	35.5	28.9	60.2	52.8	68.3	61.6	61.9	46.2

^*^The prevalence differs from Table 2 since one observation can solely exclusively belong to one mistreatment form ((1) solely incivility, (2) solely identity-based harassment, (3) incivility and identity-based harassment or (4) no incivility and no identity-based harassment)

Overall (except for European nurses and male physicians), physicians tended to have a higher prevalence of solely reporting identity-based harassment compared to solely reporting workplace incivility, with the reverse observed for nurses. Meanwhile, 13.8% of the physicians and 17.5% of the nurses experienced both workplace incivility and identity-based harassment.

19% of female physicians experienced identity-based harassment, and the prevalence was almost the same for nurses and physicians with non-European backgrounds. Additionally, 17.3% and 20.4%, respectively, experienced both identity-based harassment and workplace incivility.

The prevalence of workplace incivility was around 18% for both male and female nurses and 28% among nurses with a European background. The co-occurrence of workplace incivility and identity-based harassment was highest among female nurses (17.4%), along with nurses born outside of Europe (39.7%).

Concerning specific types of identity-based harassment and co-occurrence (Supplemental Table A3), ethnicity-based harassment was more apparent for individuals born outside of Sweden when co-occurring with other forms of mistreatment. For instance, 15.1% of nurses and 8.2% of physicians born outside of Europe experienced a combination of ethnicity-based harassment and workplace incivility. Additionally, nearly 9% of nurses born outside of Europe reported experiencing all four types of mistreatment (workplace incivility, ethnicity- and gender-based and sexual harassment) simultaneously.

## Discussion

This study aimed to understand workplace mistreatment among nurses and physicians in Sweden. We found that nearly half of the study participants experienced some type of mistreatment, with workplace incivility being the most common, regardless of the stratification category. In general, a larger share of physicians reported experiencing identity-based harassment, and a larger share of nurses reported workplace incivility. Also, the co-occurrence of workplace incivility and identity-based harassment was common. Individuals born outside of Europe faced higher rates of interpersonal mistreatment, especially ethnicity-based harassment. The identified perpetrators varied by mistreatment type.

The first key finding of this study is that workplace incivility was most often perpetrated by co-workers, while identity-based harassment more often stemmed from patients or their relatives. The second key finding is that the formal hierarchies do not always protect those in high positions from mistreatment and that the status of the profession may be exceeded by other characteristics related to social structures (I.e., sex and birth country). The third key finding indicates that mistreatment types often co-occur, particularly with workplace incivility. Social structures, operationalised as categories of combinations of profession, sex, and birth country, played a role in mistreatment experiences across all types.

### Experience and perpetrator

In our study of nurses and physicians, nearly half of the study sample had experienced some form of mistreatment in the past year (notable in Table 3). This is consistent with previous research by Velin et al. [15] focusing on final-year medical students in Sweden and highlighting that healthcare professionals often face discrimination in their work. They found that younger, less experienced healthcare professionals are exposed to more mistreatment than older, more experienced professionals [15]. Our findings revealed that nurses, female healthcare professional, and healthcare professionals born outside of Europe were the most frequently exposed to mistreatment. This corroborates prior studies focusing on either ethnicity-based harassment or racism in the healthcare sector [18, 37], discrimination within the workplace in general [10, 38] or gender-based workplace mistreatment [25].

The most prevalent type of mistreatment in our study was workplace incivility (notable in Tables 2 and 3). Also, it was more common for nurses to experience either solely workplace incivility or a combination of workplace incivility and identity-based harassment compared to physicians. Workplace incivility is characterised by its discreet and low-intensity nature, making it “easier” to perpetrate but also challenging for organisations to address [10]. Despite its subtle nature, studies consistently link workplace incivility to severe adverse health outcomes [39]. Our study highlights that over 30% of the nurses and physicians who reported workplace incivility experienced it from internal sources, i.e., from someone working within the healthcare sector (notable in Table 2). These results could be attributed to the situational influence of workplace incivility [11], i.e., that a stressful workplace creates a more hostile environment with limited time for diplomacy. A healthcare setting, which is often embossed by stress, may thus be more prone to produce workplace incivility, especially between co-workers. More specifically, different work settings, such as private versus public hospitals, present distinct stressors and patient demographics [40, 41]. This can influence interactions between healthcare workers and patients and interactions among the employees themselves. Variations in work settings require further investigation, as these differences may impact the prevalence and experience of workplace mistreatment, suggesting the need for tailored interventions based on the specific context being studied.

Our study also confirms that social structures based on sex, birth country and profession may influence the risk of interpersonal mistreatment (Supplemental Table A4). These social structures had a greater impact on the experience of identity-based harassment compared to workplace incivility. Previous studies argue that workplaces with hierarchical structures based on hierarchical status [13], such as the healthcare sector, are linked to increased experience of mistreatment, where a higher status is protective against experiences of mistreatment [1, 32]. However, the formal hierarchies of the profession, which are argued to protect from mistreatment, may be exceeded by other hierarchies based on social structures. Our study shows that when regarding the exclusive experience of identity-based harassment (Table 3), physicians report a higher prevalence than nurses. Additionally, female physicians experienced more identity-based harassment than male physicians. This difference in prevalence between female and male physicians could be explained by the norms of working as a physician, which historically has been viewed as a male-coded profession [32].

In terms of identity-based harassment, our results indicate that independent of the stratified category, it was most common to be mistreated by an external perpetrator (notable in Table 2). These findings are concerning, especially in light of previous research indicating that healthcare professionals tend to normalise harassment when it originates from patients or patients’ relatives [18, 28, 32, 37]. Such normalisation often occurs as a coping mechanism or by attributing the mistreatment to the stressful circumstances patients or relatives might be experiencing [18]. Further, in industries with a high prevalence of harassment, employees tend to identify fewer situations as harassment, which may lead to underreporting [42].

### Vulnerable groups

In examining specific stratified categories, two groups stood out for experiencing notable levels of mistreatment: *nurses born outside of Europe and female physicians*. These findings align with research by Velin et al. [15], which found that discrimination was more prevalent among medical students who are women and among those born outside Scandinavia.

Despite equal gender composition among physicians, women in this occupational group still face substantial prejudice and bias related to their gender [32]. Our study revealed that nearly one-third of *female physicians* experienced gender-based harassment within the past year (Supplemental Table A2). This aligns with the notion that the medical profession, although equally gender-diverse, is still predominantly perceived as male-dominated, with 'masculine' characteristics defining it [10]. Women often constitute a minority in many specialities, contributing to an internal sex division within the profession [21]. Consequently, being a minority or viewed as breaking the normative gender characteristics for the profession might increase the risk of being exposed to mistreatment. This poses concerns not only regarding the mental health impact of experiencing discriminatory acts but also the increased risk of identity-based harassment and intentions to leave the profession, potentially leading to a less equitable workplace and loss of competence [10].

*Nurses born outside of Europe* reported the second-highest prevalence of any form of mistreatment (notable in Tables 2 and 3). Our results suggest that his rather small group (3.5% of the nurses) is experiencing a work environment in health care ripe with hostile behaviours. Particularly, ethnicity-based harassment stood out, with over half of the nurses born outside of Europe having experienced ethnicity-based harassment within the last year (Supplemental Table A2). These results are in line with those of earlier studies about healthcare professionals’ experiences of different forms of harassment based on ethnicity or race [18, 37]. However, in their meta-analysis, McCord et al. [25] conclude that sex and race differences in perceived workplace mistreatment had decreased over time. However, differences between stratified categories have increased (ibid.). Comparable with our results, when reviewing stratified categories, the prevalence differed substantially, highlighting the importance of reporting workplace mistreatment for different social and organisational groups.

Moreover, previous studies have demonstrated that *nurses born outside of Europe* are predominantly exposed by external perpetrators, i.e. patients and their relatives [15, 37]. It has been pointed out that when it comes to ethnic minority nurses, there is a higher risk of being exposed to sexual harassment, uncivil behaviour, verbal and physical assaults, and ethnic discrimination from patients and their relatives [15, 18, 37]. Likewise, the results of our study show that a large proportion of nurses, especially nurses born outside of Europe, have been highly exposed to external perpetrators regarding all types of mistreatments (Table 2 & Supplemental Table A2). However, a large proportion of healthcare professionals are also experiencing harassment from their co-workers. Over 30% of those exposed (nurses and physicians) had experienced workplace incivility from someone at work, while nearly 15% of the exposed nurses born outside of Sweden had experienced identity-based harassment from co-workers (Table 2). While research highlights external perpetrators within the healthcare sector [18], our study underscores significant concerns within the internal social work environment as well. In health care, there is also a risk of underreporting mistreatment by co-workers due to the risk of breaking the codes of disciplinary collegiality and loyalty [34]. This warrants further research on how to improve the work environment and decrease workplace incivility and harassment, as well as the effects on individual well-being and patient care quality.

### Co-occurrence of interpersonal mistreatment

Our study reveals that various forms of mistreatment tend to co-occur; however, this co-occurrence varies across stratified categories of healthcare professionals. Our findings align with previous studies indicating that harassment is often connected and experienced with other forms of interpersonal mistreatment [9, 12]. This study highlights the need for future studies that investigate different types of interpersonal mistreatment both separately and in overarching concepts.

## Limitations

This study’s major strength is that it utilises cross-sectional data from a large and representative sample of nurses and physicians, enabling us to conclude about the broader healthcare population. However, this approach has several limitations, as described below.

This study contains a large amount of descriptive data, which is, to some extent, hard to grasp. However, this is the first time this type of data has been combined in one study, analysing two different professions, physicians and nurses, their experiences of four different types of mistreatments, their perpetrator, and how these types of mistreatments co-occur, along with differences concerning sex and birth country. This study, therefore, makes an important contribution to current knowledge and is a key publication for future studies.

Regarding the measurement of identity-based harassment, including ethnicity-based harassment, gender-based harassment, and sexual harassment, they are largely interrelated, where one type of harassment is hard to separate from the other. For example, to know whether you have been harassed due to your sex or with the allusion to your sexuality can be hard to ascertain in a situation of inappropriate comments or physical touch. Thus, since the prevalence of the different types of harassment in each survey (nurses and physicians) was measured using one item per harassment, the actual harassment in mind might not have been measured. Although each survey question was defined, specifying the type of harassment and its characteristics, there could have been difficulties knowing what type of harassment you have been exposed to. Furthermore, as other forms of workplace mistreatment, such as discrimination based on sexuality, religion, or age, often overlap with the types of identity-based harassment researched in this study, more research is needed. Given the challenges in distinguishing between different kinds of mistreatment, research combining qualitative and quantitative methods is crucial to better understand how nurses and physicians perceive and navigate these complexities of workplace mistreatment.

Furthermore, the high prevalence of individuals not stating their perpetrator regarding identity-based harassment (36.1%) is troublesome, with a high amount of missing individuals (Table 2). Possibly, there are other perpetrators who were not captured by the items in the LOHHCS dataset. However, only 5% did not state the perpetrator of workplace incivility, which makes us question if there are other reasons in play. Perhaps there is a concern for fear of retaliation, although the survey is anonymous. Nonetheless, there is a gap between the number who stated experiencing identity-based harassment but then not wanting to state their perpetrator, highlighting a need for further studies exploring this relation between type of perpetrator and harassment within the hierarchical context of medicine.

Acknowledging one’s victimisation can be challenging [28], and some people may be more prone to self-label experiences as mistreatment than others. Thus, the results of this study need to be viewed as self-identified and labelled experiences, which may differ from the objective occurrence of potentially offensive behaviours.

## Practical implication

Our study suggests variation in experiences of mistreatment among healthcare workers. Existing research has demonstrated the association between these experiences and negative health outcomes, such as heightened utilisation of psychotropic medications and increased rates of burnout, depression and suicide [3–8]. The healthcare sector confronts significant challenges related to increasing demands and struggles in recruiting and retaining trained staff [43, 44]. Along with previous studies about healthcare professionals [1, 18, 22], this study recognises that certain individuals, due to social structures, may face increased exposure to interpersonal mistreatment at work. These challenges arise both internally within the organisations and from external perpetrators. Additionally, in line with previous research [15, 18], this study underscores the need for organisations to improve the integration of migrant workers into the Swedish healthcare sector, and antiracist interventions that prevent ethnicity-based harassment and discrimination. The need for preventive measures also applies to female doctors, who should not be subjected to discrimination. In compliance with legal obligations of the EU (Directive 89/391/EEC), healthcare organisations must actively address and mitigate these concerns to ensure staff retention, ultimately contributing to enhanced patient safety. Future interventions to prevent mistreatment in healthcare should focus on both organisational structures and individuals responsible for harmful actions.

## Conclusion

Our study explored mistreatment among healthcare professionals in Sweden, highlighting patterns based on sex, birth country, and professional role. Workplace incivility and identity-based harassment varied within and between professions, with co-workers being the primary source of workplace incivility and patients and their relatives more commonly causing identity-based harassment. Notably, the hierarchical status may not shield physicians from mistreatment, as social structure can supersede professional standing. In conclusion, mistreatment types often co-occur, influenced by profession, sex, and birth country. To address the issues highlighted in this study, understanding the context, individual vulnerability, and co-occurrence is crucial for informed interventions and future research.

## Supplementary Information

Supplementary Material 1.

## Data Availability

The datasets analysed during the current study are not publicly available due to the General Data Protection Regulation, the Swedish law SFS 2018:218, the Swedish Data Protection Act, the Swedish Ethical Review Act and the Public Access to Information and Secrecy Act but are available from the corresponding author on reasonable request.

## Abbreviations

WI: Workplace Incivility
EBH: Ethnicity-Based Harassment
GBH: Gender-Based Harassment
SH: Sexual Harassment
LOHHCS: Longitudinal Occupational Health Survey for Health Care in Sweden
ICC: Interclass correlation
