# Supplementary material for: Workplace mistreatment of Swedish health care professionals: prevalence and perpetrators across profession, sex, and birth country
Source: BMC Health Serv Res. 2025 Mar 29;25:465. doi: 10.1186/s12913-025-12620-0 (PMC11954179; doi:10.1186/s12913-025-12620-0)
Supplement: Supplementary file 1 — Supplementary Material 1. [file 12913_2025_12620_MOESM1_ESM.docx]

**Appendix: Supplemental material Table A1, A2, A3 and A4**

| Supplemental Table A1: Collected survey sample of demographics by profession | | | |
| --- | --- | --- | --- |
|  | **Total** | **Nurses** | **Physicians** |
|  | **Count(%)** | **Count(%)** | **Count(%)** |
| Number of | 5615(100) | 2903(51.7) | 2712(48.3) |
| Sex | | | |
| Women | 4161(74.1) | 2615(90.1) | 1546(57.0) |
| Men | 1451(25.9) | 288(9.9) | 1166(43.0) |
| Country of birth | | | |
| Sweden | 4824(86.6) | 2641(91.7) | 2183(81.2) |
| Within Europe | 499(9.0) | 148(5.1) | 351(13.1) |
| Outside Europe | 246(4.4) | 92(3.1) | 154(5.7) |
| Sex by birth country | | | |
| Sweden women | 3622(65.0) | 2381(82.6) | 1241(46.2) |
| Sweden men | 1202(21.6) | 260(9.0) | 942(35.0) |
| Europe women | 354(6.4) | 137(4.8) | 217(8.1) |
| Europe men | 145(2.6) | 11(0.4) | 134(5.0) |
| Non-Europe women | 151(2.7) | 80(2.8) | 71(2.6) |
| Non-europe men | 95(1.7) | 12(0.4) | 83(3.1) |
| Seniority | | | |
| >15y | 2666(47.6) | 1552(53.6) | 1114(41.2) |
| 11-15y | 939(16.8) | 452(15.6) | 487(18.0) |
| 5-10y | 974(17.4) | 417(14.4) | 557(20.6) |
| <5y | 1021(18.2) | 474(16.4) | 547(20.2) |

| Supplemental Table A2: Prevalence and perpetrator of mistreatment across profession, sex and ethnicity (%) | | | | | | | | | | | | | | |
| --- | --- | --- | --- | --- | --- | --- | --- | --- | --- | --- | --- | --- | --- | --- |
|  |  |  | **Nurses** | | | | | | **Physicians** | | | | | |
|  |  |  |  | Sex | | Birth Country | | |  | Sex | | Birth Country | | |
|  |  | **Total (%)** | **Total nurses (%)** | Women (%) | Men (%) | Sweden (%) | Within Europe (%) | Outside Europe (%) | **Total physicians (%)** | Women (%) | Men (%) | Sweden (%) | Within Europe (%) | Outside Europe (%) |
| Workplace Incivility | **Incivility last 12 months** | 29.5 | 34.4 | 35.6 | 33.1 | 32.9 | 50.0 | 53.3 | 25.0 | 26.5 | 23.1 | 23.6 | 28.7 | 37.6 |
|  | **Perpetrator*** | | | | | | | | | | | | | |
|  | Not stated | 5.0 | 4.5 | 4.5 | 4.1 | 4.1 | 4.5 | 10.8 | 7.0 | 6.2 | 7.9 | 7.4 | 2.0 | 13.9 |
|  | Solely internal exposure | 32.1 | 30.9 | 32.6 | 17.5 | 29.8 | 40.2 | 31.8 | 36.5 | 40.7 | 31.1 | 32.6 | 44.5 | 42.1 |
|  | Solely external exposure | 8.1 | 8.1 | 7.7 | 11.2 | 7.8 | 8.7 | 13.5 | 8.3 | 5.4 | 12.2 | 8.9 | 8.1 | 5.1 |
|  | Internal and external exposure | 54.8 | 56.6 | 55.3 | 67.1 | 58.3 | 46.5 | 43.9 | 48.2 | 47.7 | 48.8 | 51.1 | 45.4 | 38.8 |
| Ethnicity Based Harassment | **Ethnicity- Based Harassment last 12 months** | 19.8 | 5.1 | 4.3 | 6.7 | 2.5 | 19.3 | 50.1 | 8.5 | 7.6 | 9.4 | 3.7 | 15.4 | 30.6 |
|  | **Perpetrator*** | | | | | | | | | | | | | |
|  | Not stated | 7.0 | 8.4 | 8.8 | 5.9 | 17.2 | 4.7 | 0.0 | 4.9 | 4.4 | 5.3 | 9.5 | 0.7 | 3.0 |
|  | Solely internal exposure | 18.6 | 17.0 | 17.9 | 12.0 | 12.2 | 36.1 | 13.8 | 21.2 | 19.7 | 22.4 | 18.2 | 21.6 | 24.9 |
|  | Solely external exposure | 52.5 | 57.0 | 53.6 | 76.2 | 62.5 | 47.6 | 55.9 | 45.2 | 41.6 | 48.3 | 58.4 | 35.9 | 41.9 |
|  | Internal and external exposure | 21.9 | 17.6 | 19.7 | 5.9 | 8.2 | 11.5 | 30.2 | 28.7 | 34.3 | 24.0 | 13.9 | 41.7 | 30.2 |
| Gendered Based Harassment | **Gendered Based Harassment last 12 months** | 15.6 | 19.8 | 20.2 | 16.8 | 19.5 | 21.0 | 28.0 | 19.6 | 30.1 | 8.3 | 20.2 | 18.2 | 17.4 |
|  | **Perpetrator*** | | | | | | | | | | | | | |
|  | Not stated | 82.0 | 83.5 | 83.7 | 81.5 | 89.1 | 50.5 | 21.6 | 78.0 | 82.0 | 62.4 | 88.4 | 56.0 | 29.3 |
|  | Solely internal exposure | 4.4 | 4.2 | 4.4 | 2.4 | 2.9 | 20.0 | 11.2 | 4.8 | 3.8 | 8.8 | 3.3 | 10.5 | 7.0 |
|  | Solely external exposure | 9.4 | 9.6 | 9.1 | 13.8 | 6.9 | 23.6 | 39.3 | 9.1 | 6.5 | 19.2 | 6.0 | 12.3 | 32.6 |
|  | Internal and external exposure | 4.2 | 2.8 | 2.8 | 2.4 | 1.0 | 5.9 | 27.8 | 8.0 | 7.7 | 9.5 | 2.4 | 21.1 | 31.2 |
| Sexual harassment | **Sexual Harassment last 12 months** | 6.0 | 17.5 | 18.0 | 14.1 | 17.4 | 16.3 | 21.8 | 10.3 | 12.9 | 7.5 | 11.8 | 5.5 | 11.2 |
|  | **Perpetrator*** | | | | | | | | | | | | | |
|  | Not stated | 16.0 | 14.9 | 13.6 | 27.4 | 14.3 | 9.5 | 29.3 | 21.2 | 23.1 | 17.8 | 21.0 | 19.8 | 25.9 |
|  | Solely internal exposure | 11.7 | 9.5 | 8.5 | 20.0 | 9.9 | 8.7 | 4.5 | 21.4 | 15.9 | 31.6 | 20.6 | 28.9 | 20.1 |
|  | Solely external exposure | 62.3 | 65.4 | 67.7 | 42.7 | 66.3 | 70.3 | 45.9 | 48.4 | 54.5 | 37.0 | 48.6 | 47.0 | 45.9 |
|  | Internal and external exposure | 10.0 | 10.2 | 10.3 | 9.9 | 9.5 | 11.5 | 20.2 | 9.0 | 6.5 | 13.7 | 9.8 | 4.3 | 8.1 |
| * Of those with >= 1 experience in the past 12 months. | | | | | | | | | | | | | | |

| Supplemental Table A3: Isolated experience of mistreatment in total divided by profession and sex/birth group * | | | | | | | | | | | | | |
| --- | --- | --- | --- | --- | --- | --- | --- | --- | --- | --- | --- | --- | --- |
|  |  | **Nurses** | | | | | | **Physicians** | | | | | |
|  |  |  | Sex | | Birth Country | | |  | Sex | | Birth Country | | |
| Mistreatment form | **Total experience (%)** | **Total nurses (%)** | Men (%) | Women (%) | Sweden (%) | Within Europe (%) | Outside Europe (%) | **Total physicians (%)** | Men (%) | Women (%) | Sweden (%) | Within Europe (%) | Outside Europe (%) |
| Workplace Incivility (WI) | 16.8 | 18.6 | 18.6 | 18.5 | 18.2 | 28.3 | 12.7 | 12.0 | 13.3 | 10.8 | 12.3 | 10.5 | 14.0 |
| Ethnicity Based Harassment (EBH) | 1.2 | 0.8 | 1.1 | 0.8 | 0.3 | 4.3 | 8.0 | 2.1 | 2.7 | 1.5 | 0.6 | 4.2 | 8.9 |
| Gender-Based Harassment (GBH) | 4.2 | 3.5 | 3.0 | 3.6 | 3.6 | 3.5 | 1.5 | 6.0 | 2.0 | 9.8 | 6.9 | 4.6 | 1.0 |
| Sexual Harassment (SH) | 3.2 | 3.5 | 2.7 | 3.6 | 3.6 | 3.2 | 2.5 | 2.3 | 2.4 | 2.2 | 2.6 | 1.4 | 2.0 |
| WI+EBH | 1.6 | 1.3 | 1.7 | 1.2 | 0.5 | 4.3 | 15.1 | 2.4 | 3.4 | 1.4 | 1.2 | 3.7 | 8.2 |
| WI+GBH | 4.5 | 4.6 | 4.7 | 4.6 | 4.7 | 3.6 | 2.1 | 4.4 | 1.3 | 7.2 | 4.8 | 3.7 | 1.2 |
| WI+SH | 2.4 | 2.9 | 3.1 | 2.8 | 3.1 | 1.7 | 0.0 | 1.2 | 1.4 | 1.1 | 1.4 | 0.2 | 2.1 |
| EBH+GBH | 0.4 | 0.2 | 0.6 | 0.2 | 0.1 | 0.7 | 2.4 | 0.9 | 0.3 | 1.4 | 0.4 | 0.7 | 4.9 |
| EBH+SH | 0.1 | 0.1 | 0.0 | 0.1 | 0.0 | 1.0 | 1.6 | 0.0 | 0.0 | 0.0 | 0.0 | 0.0 | 0.0 |
| GBH+SH | 2.8 | 2.9 | 1.2 | 3.1 | 3.2 | 0.0 | 0.0 | 2.6 | 1.0 | 4.0 | 3.2 | 0.9 | 1.6 |
| WI+EBH+GBH | 1.0 | 0.7 | 0.7 | 0.7 | 0.3 | 3.6 | 7.6 | 1.6 | 1.2 | 2.0 | 0.4 | 4.9 | 4.1 |
| WI+GBH+SH | 5.3 | 6.3 | 4.6 | 6.5 | 6.6 | 5.1 | 2.3 | 2.8 | 0.9 | 4.4 | 3.5 | 1.4 | 0.5 |
| WI+EBH+SH | 0.2 | 0.3 | 0.5 | 0.2 | 0.1 | 0.8 | 3.8 | 0.1 | 0.2 | 0.0 | 0.1 | 0.0 | 0.7 |
| EBH+GBH+SH | 0.3 | 0.4 | 0.7 | 0.3 | 0.3 | 0.6 | 2.7 | 0.2 | 0.2 | 0.2 | 0.2 | 0.1 | 1.0 |
| WI+EBH+GBH+SH | 1.4 | 1.4 | 1.6 | 1.4 | 0.9 | 4.0 | 8.8 | 1.3 | 1.4 | 1.2 | 0.8 | 1.9 | 3.6 |
| No experience | 54.7 | 52.6 | 55.4 | 52.2 | 54.5 | 35.5 | 28.9 | 60.2 | 68.3 | 52.8 | 61.6 | 61.9 | 46.2 |
| *One observation can solely belong to one category of mistreatment form | | | | | | | | | | | | | |

| Supplemental Table A4:  Multi-mixed model analysis: Workplace Incivility and Identity Based Harassment sorted by sex with birth country (M1) and age (M2) | | | | |
| --- | --- | --- | --- | --- |
| Value | **WI** | | **Identity-Based Harassment** | |
|  | *M1* | *M2* | *M1* | *M2* |
| Int. | 0.79* | 0.60* | 0.44* | 0.25* |
| Age |  |  |  |  |
| *23-35* |  | 0.39* |  | 0.44* |
| *36-44* |  | 0.23* |  | 0.20* |
| *45-56* |  | 0.14* |  | 0.09* |
| *57-76* |  | 0 |  | 0 |
| Resid. | 0.418* | 0.398* | 0.355* | 0.327* |
| Inters. | 0.024* | 0.026* | 0.041* | 0.048* |
| Log.2. | 271302.989 | 264543.862 | 251426.097 | 240020.452 |
| ICC | 5.4% | 6.2% | 10.4% | 12.7% |

**P-value <.05*
